# Optimized *Ginkgo biloba* extract EGb 761^®^: boosted therapeutic benefits with minimized CYP enzyme interference

**DOI:** 10.3389/jpps.2025.14614

**Published:** 2025-08-26

**Authors:** Sunbeom Kwon, Suji Jeong, Seulah Lee

**Affiliations:** 1 Department of Convergent Biotechnology and Advanced Materials Science, College of Life Sciences, Kyung Hee University, Yongin, Republic of Korea; 2 BK21 Interdisciplinary Program in IT-Bio Convergence System, Kyung Hee University, Yongin, Republic of Korea

**Keywords:** EGb 761^®^, protocatechuic acid, cognitive enhancement, molecular docking, CYP inhibition

## Abstract

**Object:**

The development of cognitive-enhancing drugs from *Ginkgo biloba* extract is actively pursued worldwide. This study compares the chemical compositions of different *G. biloba* extracts and their formulated drugs, highlighting the distinguishing characteristics and potential benefits of optimized *G. biloba* extract, EGb 761^®^.

**Methods:**

We analyzed three *G. biloba* extracts and fifteen formulated drugs using HPLC, principal component analysis, and LC-MS/MS to identify key compositional differences. Molecular docking analysis was conducted to evaluate the binding affinity of the key component with a target protein involved in cognitive enhancement. CYP inhibition assays were performed on selected extracts and their derived products to examine drug-drug interactions.

**Results:**

EGb 761^®^ and its formulated drugs displayed a unique composition, characterized by a significantly higher level of protocatechuic acid (PCA). PCA demonstrated strong interactions with the M_1_ receptor, acetylcholinesterase, glycogen synthase kinase-3, which are the key targets for cognitive enhancement. CYP inhibition assays indicated that EGb 761^®^ and the drugs derived from EGb 761^®^ had lower inhibitory activity compared to other samples.

**Conclusion:**

The high PCA content in EGb 761^®^ may contribute to cognitive benefits. With low CYP inhibition, it suggests minimal interference with drug metabolism, highlighting its potential as a safer cognitive enhancer. Ultimately, this study indicates that the composition of EGb 761^®^ can be effectively leveraged for its pharmacological benefits.

## Introduction

Cognitive enhancement typically involves interventions that directly influence brain function, such as pharmaceutical drugs or brain stimulation techniques [1]. It is regarded as an essential approach for addressing cognitive dysfunction. Cognitive dysfunction constitutes a significant and inadequately managed aspect of psychiatric disorders, disrupting patients’ social and occupational functionality. This highlights the urgent need for advanced therapeutic strategies to tackle the problem [2]. Besides aging and Alzheimer’s disease, which are well-recognized causes of cognitive dysfunction, psychiatric disorders such as depression, schizophrenia, anxiety and bipolar disorders are also reported to contribute to cognitive impairment. These conditions manifest in various forms of cognitive dysfunction, including impaired decision-making, emotional bias, attention deficits, and diminished learning and memory abilities [3]. Various synthetic drugs, including donepezil, modafinil, guanfacine, and atomoxetine, as well as natural product-derived pharmaceutical such as D-cycloserine, are utilized for the treatment of cognitive impairment. Notably, numerous studies have reported the use of plant-derived extracts from species such as *Coffea arabica*, *Camellia sinensis*, *Nicotiana tabacum*, *Melissa officinalis*, and *Ginkgo biloba* as agents for cognitive enhancement [4–6].

The leaves of *Ginkgo biloba* have been used traditionally as a medicinal plant for many years and are extensively studied due to their established therapeutic benefits for peripheral vascular and cerebrovascular conditions [6]. Particularly, research on cognitive impairment and neurodegenerative disorders, such as Alzheimer’s disease, has been actively conducted. *G*. *biloba* extracts are prescribed as nootropic agents for aging and Alzheimer’s disease in various parts of the world, including Europe [6]. The extracts of *G*. *biloba* leaves are reported to contain various secondary metabolites, including flavonoids, terpenoids, and phenolic compounds, among which numerous flavonoids and phenolic compounds exhibit antioxidant and neuroprotective effects [7]. Terpenoids, such as ginkgolides and bilobalides, are highly valued for their medicinal benefits, which include protection of hippocampal neurons, enhancement of memory and learning abilities, and mitigation of neuronal damage [7]. Countries like Germany, China, Japan, and the United States have actively participated in the development of G. biloba leaf extracts as a raw material for pharmaceutical applications [8].

One of the most well-known standardized extracts of *G*. *biloba* leaves is EGb 761^®^, developed by Dr. Willmar Schwabe Pharmaceuticals in Germany [8]. EGb 761^®^ has been utilized in Europe since the early 1980s. It contains approximately 24% flavone glycosides (primarily quercetin, kaempferol, and isorhamnetin) and about 6% terpenoid lactones (2.8–3.4% ginkgolides A, B, C, and 2.6–3.2% bilobalide) as its main active components, along with other constituents such as proanthocyanidins and organic acids [8]. It is reported to have diverse physiological effects, such as improving blood circulation, preventing platelet aggregation, providing antioxidant and neuroprotective effects, and having therapeutic potential for sudden hearing loss and tinnitus [9].

This study compares the compositional constituents of various standardized *G*. *biloba* extracts, including EGb 761^®^. It also analyzes the compositional profiles of cognitive-enhancement drugs manufactured using different *G*. *biloba* extracts. Additionally, CYP enzyme inhibition of drugs composed of different extracts was assessed to evaluate their compatibility for co-administration with other drugs, aiming to identify universally applicable raw materials and final drug products. Finally, a key component that demonstrated notable differences in content in different extracts was assessed for its binding affinity with a potential target protein using *in silico* molecular docking.

## Materials and methods

### Chemicals

Chemicals and reagents used in the chemical profiling of *Ginkgo biloba* leaf extracts include HPLC-grade acetonitrile, HPLC-grade methanol (Honeywell, NC, United States), EP grade formic acid (Duksan, Ansan, Korea) and primary reference standard grade protocatechuic acid (HWI, Rülzheim, Germany). All raw materials and final drug products were purchased from the market in Korea. All commercial products, including EGb 761^®^ (lot no. 29017682, 29017869, and 29017868), GBE1 (20210403/10932, 20220807/10935, and 20221110/10935), GBE2 (CX-210706), A1 (230009, 230010), A2 (230017, 230026, 230027, 230024, and 230040), A3 (230002, 230003), A4 (220002, 230001, 230002, and 230003), B1 (0012302, 0042302, and 0072302), B2 (0452312, 0582312), B3 (0102201, 0172301), B4 (0012301, 0052201), C1 (21001B, 21002B), D1 (20009), E1 (4001A), E2 (3038), F1 (P0001), F2 (ASC004), and F3 (GMB019) were produced by different manufactures. The reagents used in CYP inhibition assay include acetonitrile (Sigma, MO, United States), dimethyl sulfoxide (DMSO; Sigma), methanol (Duksan), α-naphthoflavone (Sigma), sulfaphenazole (Sigma), miconazole (Sigma), quinidine (Sigma), tranylcypromine (Sigma), and ketoconazole (Sigma), Vivid CYP1A2 Blue, Vivid CYP2C19 Blue, Vivid CYP2D6 Blue, Vivid CYP2E1 Blue, Vivid CYP3A4 Blue, and Vivid CYP2C9 Blue (Thermo Fisher, MA, United States). A 96-well black plate (Corning, NY, United States) was utilized for the assays, and measurements were conducted using a multimode plate reader (PerkinElmer, MA, United States).

### HPLC analysis

HPLC analysis was performed using an Arc HPLC system (Waters, Milford, MA, United States). The separation was achieved on a GL science Intersil ODS-3 (4.6 × 250 mm, 5 μm) maintained at 25°C. The mobile phase consisted of solvent A (0.1% formic acid in water) and solvent B (0.1% formic acid in acetonitrile) under a gradient program as follows: 90% solvent A and 10% solvent B at 0 min, transitioning to 75% solvent A and 25% solvent B at 54 min, 70% solvent A and 30% solvent B at 59 min, 5% solvent A and 95% solvent B from 63 to 66 min, and returning to 90% solvent A and 10% solvent B at 67 min, maintained until 75 min. The total run time was 75.0 min, with a flow rate of 1.0 mL/min. The injection volume was 10 μL, and detection was carried out at a wavelength of 270 nm. Data acquisition and processing were performed using Empower 3 software.

### Principal component analysis

Principal component analysis was performed to evaluate the variability in compound composition across all samples. The analysis was conducted using Minitab software (version 12.4.2), and results were visualized as PCA score plots to identify clustering patterns. The input data for PCA were derived from the chemical profiling results obtained by HPLC analysis of each raw material and final drug products. A total of 26 peaks with relative peak areas exceeding 1% in the chromatograms were selected. For each peak, the mean value of triplicate peak areas was calculated and used for the analysis. Detailed information on the input values used for PCA is provided in Supplementary Table S1.

### LC-MS/MS analysis

For the identification of unknown peaks, mass spectrometry (MS) analysis was conducted. Test solutions were prepared by dissolving one tablet in 100 mL of 50% MeOH. LC-MS/MS analysis was performed using deionized water (DW) and ACN (Duksan, Ansan, Korea) as solvents, with formic acid (Sigma Aldrich, MO, USA). Chromatographic separation was carried out on a Poroshell 120 EC-C18 (3.0 × 150 mm, 2.7 μm) using an Agilent 6495 LC/TQ system. The mobile phase consisted of solvent A (distilled water containing 0.1% formic acid) and solvent B (acetonitrile containing 0.1% formic acid). The gradient was linear at a flow rate of 0.6 mL/min from 10% to 25% solvent B for 54 min, from 25% to 30% B for the next 5 min, from 30% to 95% B for 7 min and finally from 95% to 10% B for another 9 min; the latter was followed by washing with methanol and re-equilibration of the column for 9 min. The analysis was conducted with an injection volume of 10 µL. Mass spectrometry was operated in both positive ([M + H]^+^) and negative ([M−H]^−^) ionization modes. While the photodiode array (PDA) detector scanned wavelengths at 270 nm, the mass spectrometer simultaneously acquired data within an *m/z* range of 150–1,500. To ensure optimal performance, the mass spectrometer was operated with a gas temperature of 250°C and a gas flow rate of 14 L/min, while the nebulizer pressure was maintained at 20 psi. Additionally, the sheath gas temperature and flow rate were set to 250°C and 11 L/min, respectively. The capillary voltage was fixed at 3000 V, and the nozzle voltage was set to 1500 V. All data were acquired and analyzed using the proprietary software provided by the instrument manufacturer.

### HR LC-MS/MS analysis

To accurately identify the main peak observed in the compound profiling analysis, high-resolution (HR) LC-MS/MS analysis was performed. Standard protocatechuic acid (PCA) and EGb 761^®^ samples were each prepared at a concentration of 10 ppm in methanol. LC was conducted using a Thermo Vanquish system coupled with a Waters Cortecs T3 column (2.1 × 150 mm, 1.6 μm particle size). The column temperature was maintained at 45°C, and the flow rate was set to 0.25 mL/min. The mobile phases consisted of solvent A (0.1% formic acid in water) and solvent B (0.1% formic acid in acetonitrile). The gradient program was applied as follows: 0–0.5 min, A:B = 97:3; 0.5–15 min, A:B = 85:15; 15–30 min, A:B = 50:50; 30–31 min, A:B = 0:100; 31–35 min, A:B = 0:100; 35–35.1 min, A:B = 97:3; 35.1–40 min, A:B = 97:3. The MS/MS analysis was carried out using a Thermo Q-Exactive Orbitrap mass spectrometer equipped with a heated electrospray ionization (H-ESI) source, operating in negative ion mode. Instrument parameters were set as follows: spray voltage, −3000 V; sheath gas flow rate, 50 Arb; auxiliary gas flow rate, 10 Arb; sweep gas flow rate, 1 Arb; ion transfer tube temperature, 320°C. The resolution settings were 35,000 for MS1 and 17,500 for MS2, with a scan range of *m/z* 100 to 1,000.

### Quantitative analysis

Quantitative analysis was conducted in triplicate using test solutions. To eliminate the differences arising from variations in drug dosage, all experimental groups were adjusted to a drug dose of 240 mg and dissolved in 100 mL of 50% MeOH. The *G. biloba* extracts were also analyzed under the same concentration conditions as the formulated drugs. The quantitative analysis of protocatechuic acid (PCA) was conducted using a standard solution prepared by dissolving a purchased standard at a concentration of 10 mg/250 mL. The analysis was conducted five times to ensure accuracy and precision. The injection volume was 10.00 µL, and the total run time for the analysis was 60.0 min. The HPLC conditions used in this analysis were identical to those described in Section *HPLC analysis*, except for the following modifications. A YMC-Triart ExRS column (4.6 × 250 mm, 5 μm) was employed, and the column temperature was maintained at 30°C. The gradient elution was programmed as follows: 0 min, A:B = 100:0; 40 min, A:B = 85:15; 45 min, A:B = 0:100; 50 min, A:B = 85:15; 58–60 min, A:B = 100:0.

### Molecular docking

The structural data of target protein (muscarinic acetylcholine receptor M1, PDB ID: 5CXV, acetylcholine esterase, PDB ID: 1EVE, *N*-methyl-D-aspartate receptor, PDB ID: 5U8C, glycogen synthase kinase-3, PDB ID: 1Q5K and *β*-secretase, PDB ID: 1TQF) and control ligand were obtained from RCSB Protein Data Bank. The chemical structures of experimental ligands were retrieved from PubChem as SMILES. The protein and ligands were optimized to perform ligand docking using Protein Preparation Workflow and LigPrep in Maestro 14.2 (©Schrödinger, Inc.). During the Protein Preparation process, all molecules other than the protein and the reported antagonist, Tiotropium, were removed, including any impurities. The receptor grid was generated using the Receptor Grid Generator tool in Maestro 14.2, with its location aligned to the reported ligand-binding site of the target protein as described in the PDB. Ligand docking was conducted to assess the binding affinity between the optimized target protein and the ligand within the defined grid space, utilizing the Extra Precision (XP) algorithm for evaluation. Following ligand docking, a minimization process, one of the tasks available in the Maestro 14.2 software, was performed to generate an optimized binding pose between the protein and ligand while minimizing the void spaces left by the removal of molecules during the Protein Preparation process. Binding energy was calculated from molecular mechanism-generalized Born surface area (MM-GBSA) tool in Maestro 14.2 to evaluate the binding affinity. The analysis was conducted with all parameters for each task set to their default setting.

### CYP enzyme activity

*Ginkgo biloba* leaf extracts (EGb 761^®^, GBE1) and final drug products (B2, B4, and A4) were dissolved in 0.1% acetonitrile at concentrations of 2.5–12.5 mg/mL. Fluorescence-based enzyme activity assays were performed using Vivid CYP enzyme substrate kits on a 96-well black plate. For each well, 40 μL of test compound solution and 50 μL of a Master Pre-Mix containing P450 BACULOSOMES Plus Reagent and Vivid Regeneration System were added. After a 10-min pre-incubation at room temperature, 10 μL of Vivid substrate and NADP^+^ were added to initiate the reactions. The plates were incubated for 40 min, and fluorescence signals were measured (Ex: 415 nm, Em: 460 nm) using a multimode plate reader. Enzyme activity was calculated as the ratio of the relative fluorescence unit (RFU) of the test compound to that of the solvent control.

### Statistical analysis

All values are presented as mean ± standard deviation (SD) and differences are statistically significant at the *p* < 0.05 level. Statistical analyses were performed using the IBM SPSS™ Statistics (Version 26.0). The statistical significance of the results was analyzed using one-way ANOVA followed by LSD *post hoc* analysis.

## Results and discussion

### Chemical profiling of *G. biloba* leaf extracts

**FIGURE 1:**
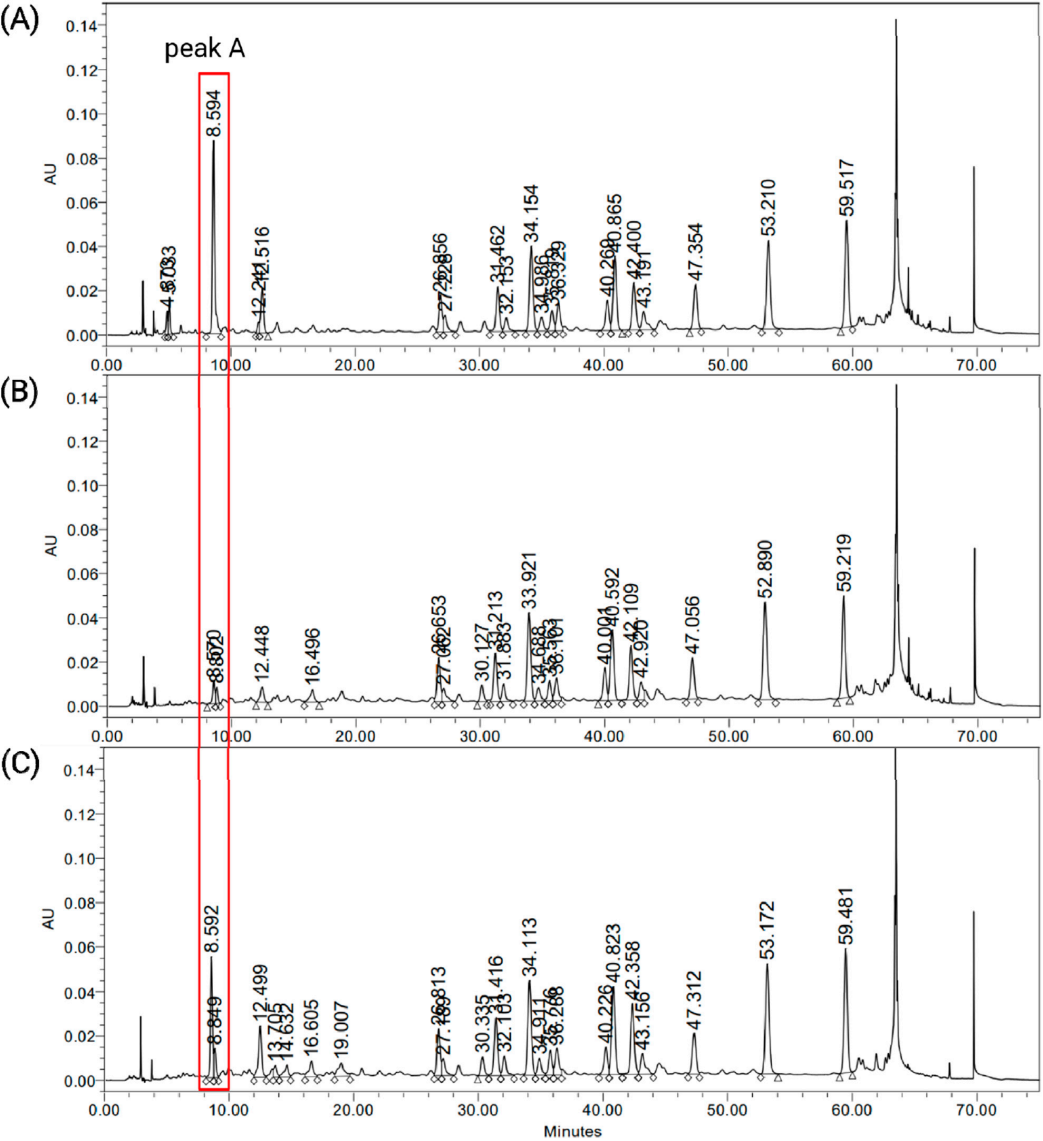
HPLC chromatograms of *G. biloba* leaf extracts, **(A)** EGb 761^®^, **(B)** GBE1 and **(C)** GBE2.

Chemical profiling was conducted on three different *G. biloba* leaf extracts, including EGb 761^®^ (Figure 1). Two other extracts were designated as GBE1 and GBE2. The results revealed that the three extracts exhibited similar patterns throughout their HPLC chromatograms under specific mobile phase conditions, except for a considerable difference near *t*_*R*_ (retention time) 8.6 min (peak A). A noticeable disparity in the area of peak A was observed, where the highest value was detected for EGb 761^®^ (1,020,871), followed by GBE2 (597,833) and GBE1 (116,679).

**FIGURE 2:**
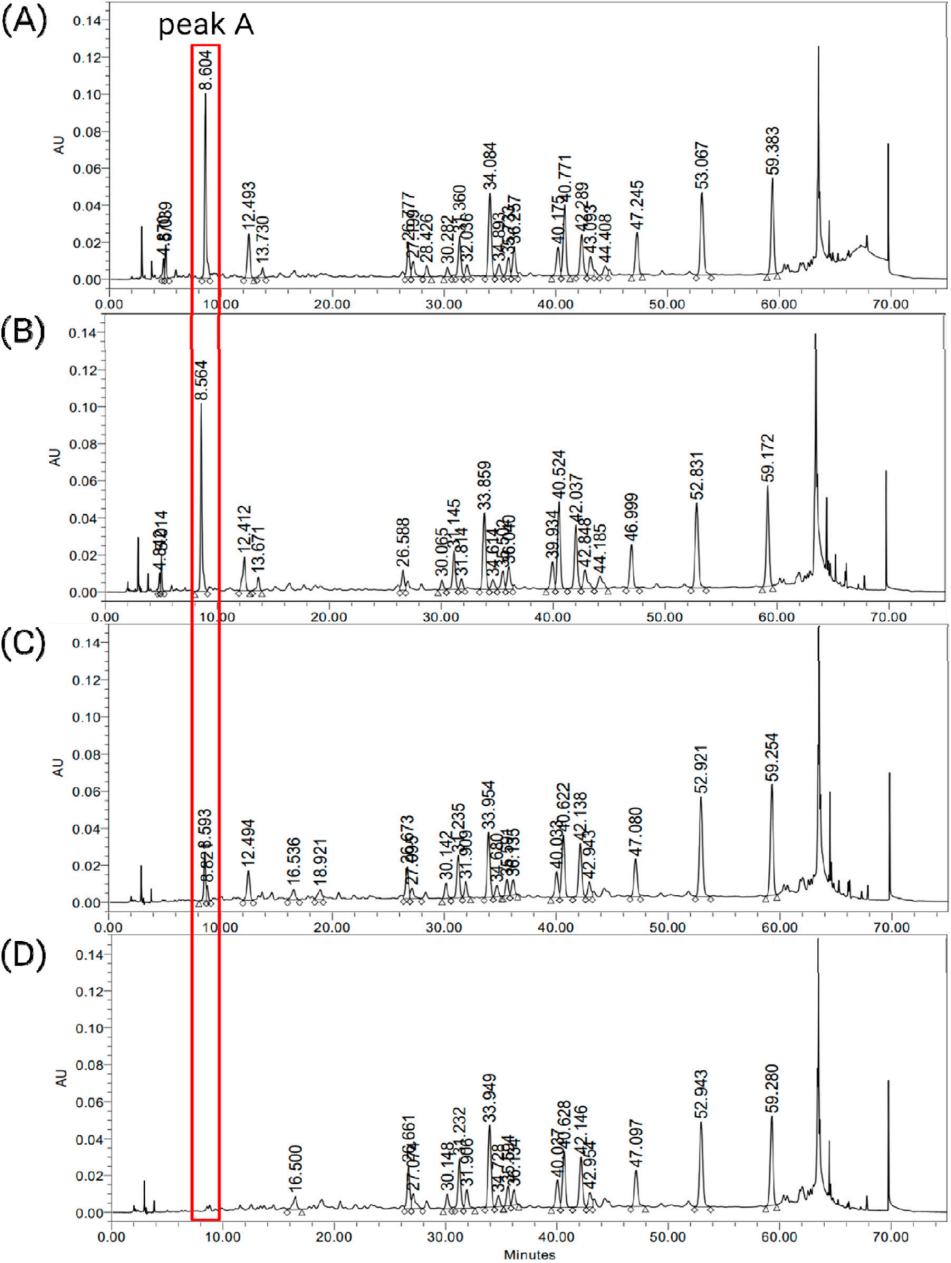
HPLC chromatograms of final drug products, **(A)** A1, **(B)** B1, **(C)** B3 and **(D)** B4.

To investigate whether the differences in peak levels observed in the raw extracts are consistently reflected in the final drug products, chemical profiling was also conducted on various final drug products (Figure 2). Drugs A1, B3, and B4 are made from raw materials EGb 761^®^, GBE2, and GBE1, respectively. B1 was produced using a proprietary raw material that was not included in this study due to its unavailability in the market. For easier interpretation and comparison of the results, the raw material used for B1 was arbitrarily designated as GBE3. As shown in Figure 2, consistent values of integrated areas of peak A, compared to those of the corresponding raw materials, were observed. B1 exhibited an integrated area value similar to that of A1. Through chemical profiling analysis of raw materials, it was confirmed that EGb 761^®^ exhibits composition that is considerably distinct from other publicly available raw materials, especially regarding peak A. Although a direct comparison between the raw materials could not be conducted, the drugs produced from EGb 761^®^ and GBE3 displayed similar chemical compositions, which may suggest a similarity between the two raw materials.

### Principal component analysis

**FIGURE 3:**
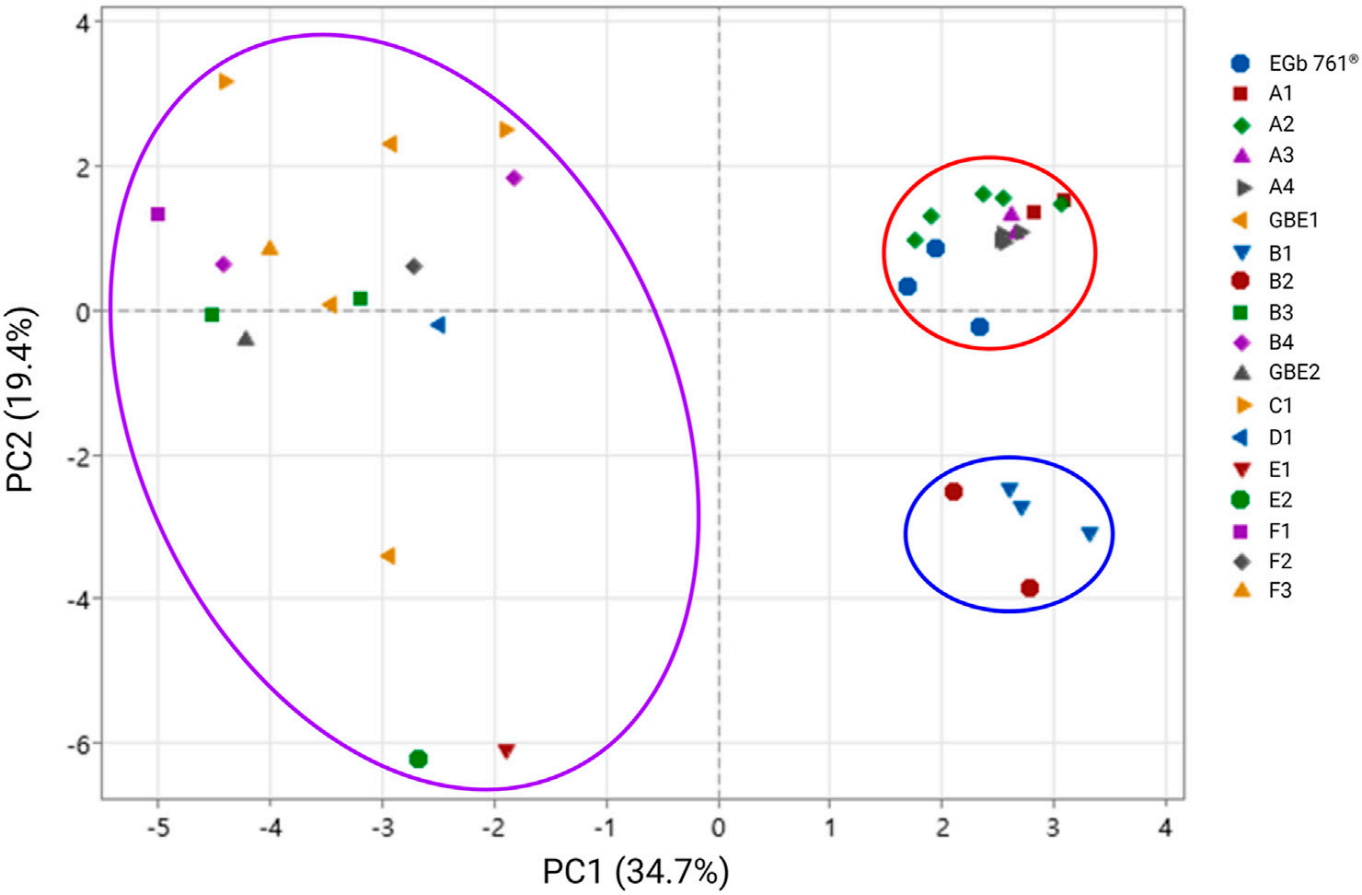
Principal component analysis of all samples. In the legend, single letters represent raw materials, while alphanumeric labels denote final products. The letters assigned to final drug products are grouped based on brand names, regardless of their corresponding raw materials, and higher numbers indicate final drug products with higher concentrations.

Principal component analysis was performed to evaluate the differences in principal components across all samples. In Figure 3, the cluster outlined in red represents raw material EGb 761^®^ and the final drug products derived from it, whereas that outlined in purple represents raw materials GBE1 and 2, as well as the corresponding final drug products. The cluster outlined in blue corresponds to final products B1 and B2, which were derived from GBE3. The cluster of EGb 761^®^ was observed to be distinctly separated from the other clusters. The results also show that the cluster for EGb 761^®^ exhibits more consistent quality compared to the clusters for raw materials GBE1 and GBE2. In general, EGb 761^®^ has a unique compound composition that is distinct from other raw materials and maintains consistent quality throughout the analysis.

### Identification of an unknown marker compound

To unambiguously identify compounds corresponding to the peaks obtained from chemical profiling analysis, LC-MS/MS was employed. Data were analyzed based on literature research and the laboratory’s internal database, allowing the assignment of peaks from the HPLC chromatogram. Information on the assigned peaks is summarized in Table 1. Most of the identified peaks corresponded to flavone glycosides, which have been reported as major constituents of EGb 761^®^ [8]. However, peak A, which exhibited the most notable difference in the compound profiling analysis, did not match any existing entries in the database. Based on molecular weight and UV pattern analysis, the peak was predicted to be protocatechuic acid (PCA), which has been predominantly reported as one of the major organic acids in *G. biloba* leaf extract [10, 11]. To confirm this, the retention time and UV pattern of PCA standard were compared with those of the profiling data. The results showed an exact match, definitively identifying the peak A as PCA (Supplementary Figures S1–S3). Furthermore, HR LC-MS/MS analysis supported this identification by demonstrating consistent molecular formula, isotopic distribution pattern, and MS/MS fragmentation profile between the PCA standard and peak A (Supplementary Figures S4–S6).

**TABLE 1:** Compound name and retention time of the assigned peaks.

Compounds	Retention time (min)
Protocatechuic acid	8.604
Quercetin-3-*O*-2″,6″-dirhamnosylglucoside	26.777
Kaempferol-3-*O*-2″,6″-dirhamnosylglucoside	31.360
Quercetin-3-*O*-rutinoside	34.084
Quercetin-2″-glucosylrhamnoside	40.175
Kaempferol-3-*O*-rutinoside	40.771
Isorhamnetin-3-*O*-rutinoside	42.289
Kaempferol-3-*O*-2″-glucosylrhamnoside	47.245
Quercetin-3-*O*-2″-(6″-*p*-coumaroyl)glucosylrhamnoside	53.067
Kaempferol-3-*O*-2″-(6″-*p*-coumaroyl)glucosylrhamnoside	59.383

### Quantitative analysis of protocatechuic acid

**FIGURE 4:**
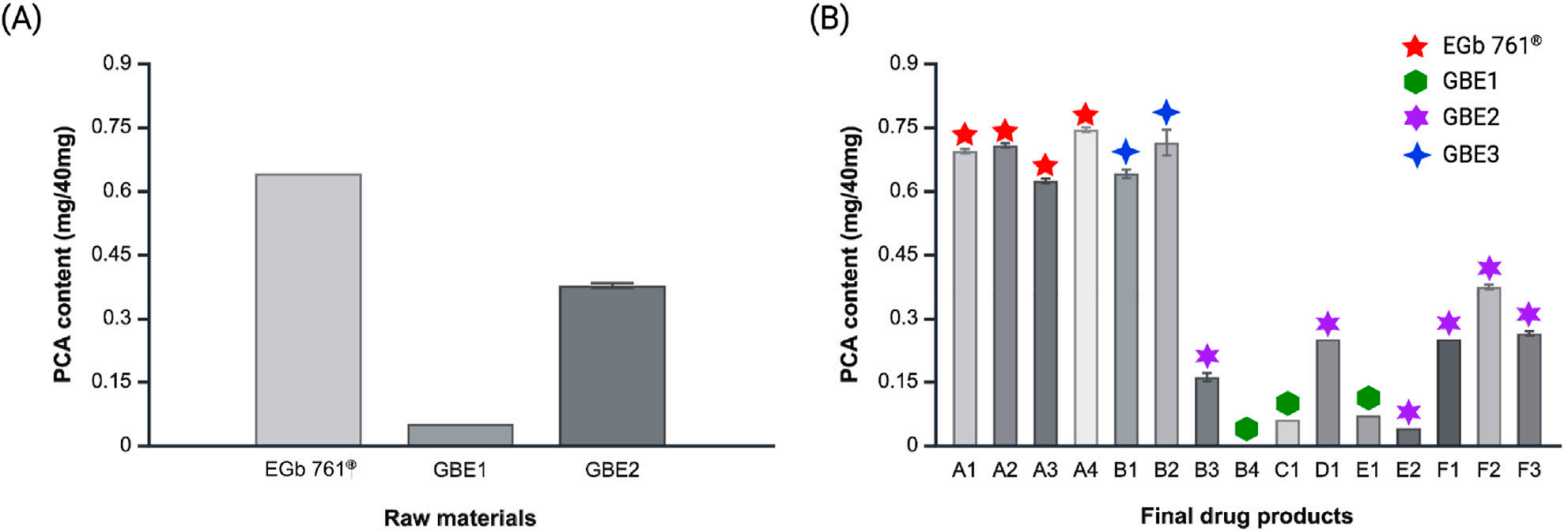
PCA contents of **(A)** raw materials and **(B)** final drug products.

The content of PCA, which exhibited the most pronounced disparity in chemical profiling and principal component analysis, was quantitatively analyzed across raw materials and final drug products (Figure 4). To eliminate potential overestimation of PCA content caused by co-eluting compounds with identical or similar retention times during quantification, the HPLC conditions were optimized to achieve maximum resolution of the PCA peak prior to analysis (Supplementary Figure S7). Among the raw materials, the PCA content was the highest in EGb 761^®^, with an average of 0.64 mg of PCA per 40 mg of the extract (Figure 4A). PCA content of 0.38 mg/40 mg and 0.05 mg/40 mg were observed for GBE2 and GBE1, respectively. In case of final drug products, similar tendency was observed, where drugs produced from EGb 761^®^, A1–A4, revealed the highest PCA content (Figure 4B). Accordingly, products derived from GBE2 generally displayed intermediate levels of average PCA content, and those manufactured from GBE1 demonstrated the lowest average PCA content. Drugs B1 and B2, which are manufactured from GBE3, showed average PCA levels similar to those of A1–A4, consistent with the results from chemical profiling and principal component analysis.

### Molecular docking

According to the results of the current study, PCA was chosen as a key component that might contribute to the distinctiveness of EGb 761^®^. Previous research has demonstrated the cognitive-enhancing effects of PCA in various animal models [12–15]. Key target proteins commonly studied in relation to cognitive enhancement include acetylcholine esterase (AChE), *N*-methyl-D-aspartate receptor (NMDA), muscarinic acetylcholine receptors (mAChRs), glycogen synthase kinase 3 (GSK-3), and *β*-secretase [16]. Previous studies have conducted molecular docking analysis of PCA, reporting its binding affinities with all the aforementioned key proteins, except for mAChRs [17–20]. mAChRs are proteins that mediate the metabolic actions of acetylcholine in the nervous system and are categorized into five subtypes, M_1_ to M_5_. Among these, the M_1_ receptor is abundantly expressed in the forebrain and is implicated in higher-order cognitive functions, such as long-term memory consolidation, learning, and spatial memory [21–23]. Thus, the binding affinity of PCA with the M_1_ receptor of mAChRs was evaluated *in silico* to further assess the impact of PCA content on the existing bioactivity of final drug products and raw materials. Since *in silico* molecular docking is subject to variability depending on the software and algorithms used, previously reported docking results were reanalyzed using the identical PDB ID from the previous studies and the docking protocol employed for the M_1_ receptor in the current study. This ensured consistency in methodology and minimized potential bias arising from software- or algorithm-specific differences.

As shown in Table 2, molecular docking analysis provided information on docking scores, binding energies, and molecular interactions between PCA and the key proteins involved in cognitive function. Among these proteins, M_1_ receptor exhibited the highest docking score, whereas *β*-secretase showed the lowest. In terms of binding energy, all protein–ligand complexes exhibited negative values, indicating energetically favorable interactions. The most stable binding energy was observed with AChE, while the interaction with NMDA receptor showed relatively less stable binding energy.

**TABLE 2:** Results of molecular docking analysis for PCA with key cognitive enhancement-related target proteins.

Target protein (PBD ID)	Docking score (kcal/mol)	Binding energy (kcal/mol)	Interaction
M_1_ receptor (5CXV)	−6.807	−28.38	H-bond: THR189, THR192, TYR381, ASN382 π-π interaction: TRP157
AChE (1EVE)	−5.728	−38.01	H-bond: ARG289, PHE288, PHE331 π-π interaction: PHE290, TRP297
NMDA receptor (5U8C)	−5.390	−16.49	H-bond: THR116, SER114, SER173 ARG121, TYR214, THR174 π-π interaction: HIE88
GSK-3 (1Q5K)	−5.554	−37.00	H-bond: VAL135, ASP133, LYS85 Salt bridge: LYS85
*β*-secretase (1TQF)	−5.383	−27.70	H-bond: GLN73, THR72

**FIGURE 5:**
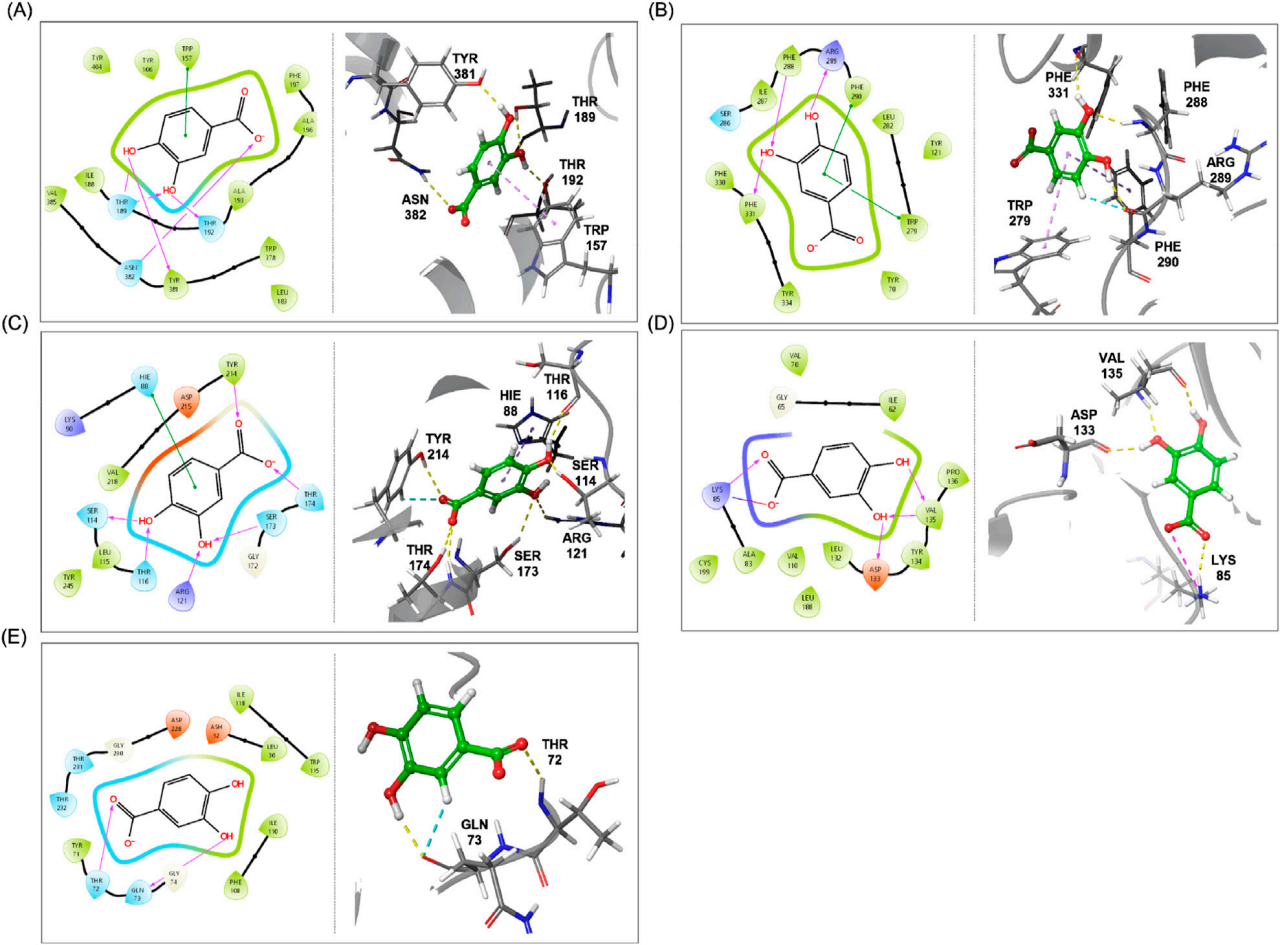
2D and 3D schemes illustrating the binding poses and interactions between PCA and key cognitive enhancement-related target proteins, **(A)** M_1_ receptor, **(B)** AChE, **(C)**, NMDA receptor, **(D)** GSK-3, and **(E)** β-secretase.

The 2D and 3D binding schemes of PCA within the binding pockets of each target protein are illustrated in Figure 5. Among the proteins analyzed, the NMDA receptor exhibited the greatest number of amino acid residues interacting with PCA. Specifically, carboxylic acid functionality of PCA formed hydrogen bonds with TYR214 and THR174 via its carbonyl oxygen and the carboxylate ion generated during the LigPrep process. In the aromatic region of PCA, 3-OH formed hydrogen bonds with ARG121 and SER173, while 4-OH interacted with SER114 and THR116. Additionally, a π–π interaction was observed between the aromatic ring of PCA and HIE88. In contrast, *β*-secretase exhibited the fewest interactions with PCA. The carbonyl oxygen formed a hydrogen bond with THR72, while 2-H and 3-OH of the aromatic ring engaged in hydrogen bonding with GLN73. Additionally, both M_1_ receptor and AChE exhibited multiple hydrogen bonds and π–π interactions with PCA. Notably, in case of GSK-3, a unique salt bridge was observed between the carbonyl oxygen of PCA and the LYS85 residue—an interaction not detected in any of the other protein targets. The molecular docking results demonstrated that PCA generally exhibits stable binding affinities and strong interactions with proteins associated with cognitive enhancement. Among the targets analyzed, M_1_ receptor, AChE, and GSK-3 showed the highest binding potential with PCA. These findings provide compelling evidence supporting previously reported cognitive-enhancing effects of PCA [12–15] and suggest that its high content may contribute positively to the development of therapeutics aimed at improving cognitive function.

### CYP enzyme activity

**FIGURE 6:**
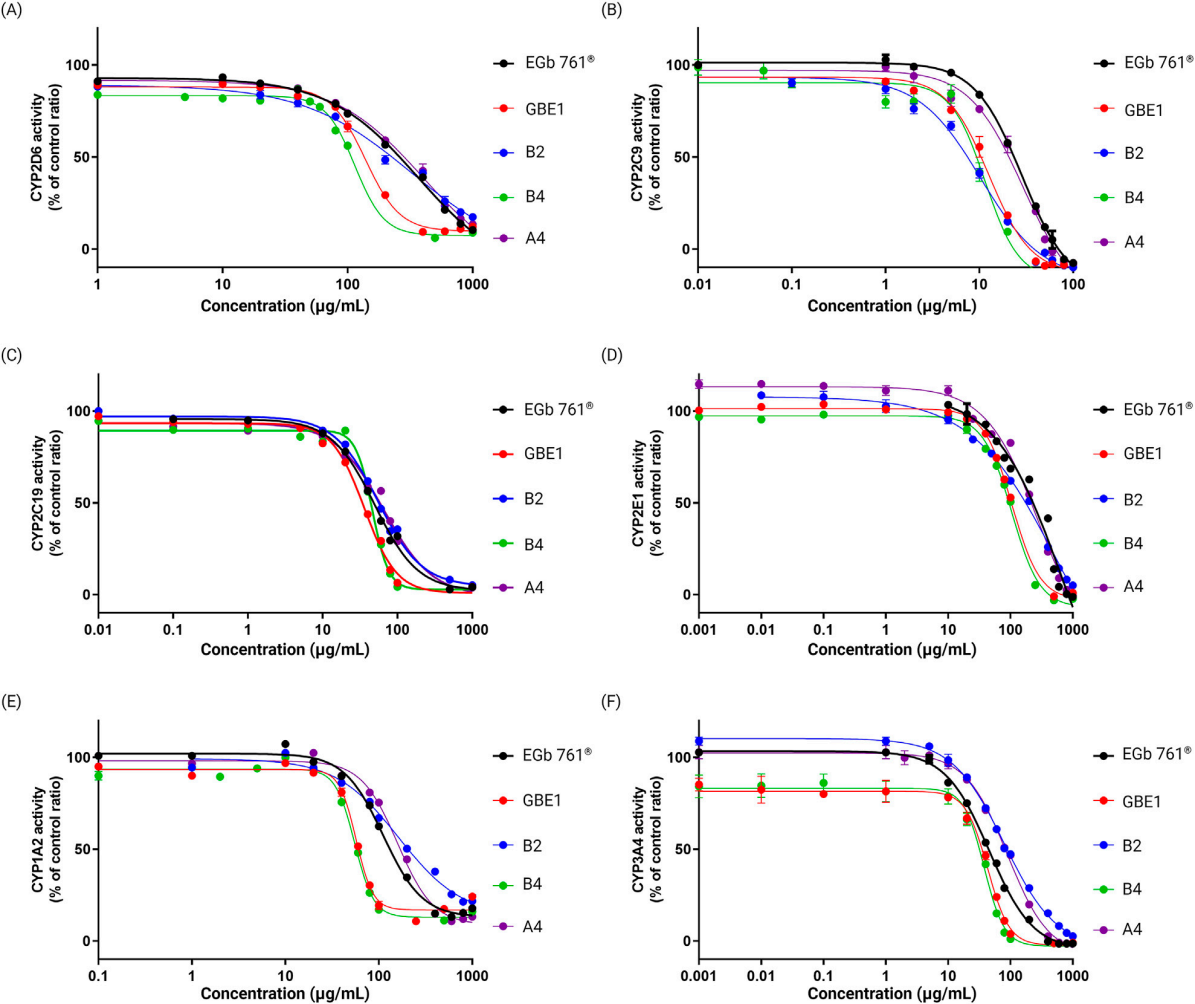
Enzymatic activity of six representative CYP enzymes, **(A)** CYP2D6, **(B)** CYP2C9, **(C)** CYP2C19, **(D)** CYP2E1, **(E)** CYP1A2, and **(F)** CYP3A4, in response to varying doses of each sample.

Patients with dementia or mild cognitive impairment (MCI) typically present with two or more comorbidities, with chronic conditions such as hypertension, diabetes, and cerebrovascular diseases being the most prevalent [24, 25]. Given this, dementia treatments and cognitive-enhancing drugs are frequently co-administered with medications for other chronic conditions, making concerns about metabolic drug-drug interactions inevitable. Studies have reported the effects of *G. biloba* leaf extracts (GBE) on cytochrome P450 (CYP) activity, that GBE may influence the metabolism of co-administered drugs [26]. In this study, we aimed to evaluate and compare the CYP inhibitory activity among different raw materials and final drug products to identify those that are more suitable for co-administration. We conducted CYP inhibitory activity assays for six representative CYP enzymes (CYP2D6, CYP2C9, CYP2C19, CYP2E1, CYP1A2, CYP3A4) using raw materials EGb 761^®^ and GBE1, which exhibited the most significant differences in previous experiments, along with three final drug products (A4, B2 and B4). Drugs A4, B2 and B4 each derive from EGb 761^®^, GBE3 and GBE1, respectively. Among them, B2 exhibited the smallest difference compared to A4 in the PCA content analyses. As shown in Figure 6, percentage of CYP activity inhibition at different concentrations of all samples were evaluated, and all samples exhibited concentration-dependent inhibitory activity in all six CYP enzymes. Overall, EGb 761^®^ and drug A4 displayed low inhibitory activity compared to GBE1 and B4 at all tested concentrations, with differences becoming more pronounced at higher concentrations. Drug B2 generally showed a pattern similar to that of EGb 761^®^ and A4; however, an exception was observed for the activity of CYP2C9, where B2 demonstrates a higher inhibition compared to GBE1 and B4. These findings suggest that EGb 761^®^ and A4 may be more suitable for co-administration as broadly applicable drug candidates compared to other raw materials and final drug products.

**FIGURE 7:**
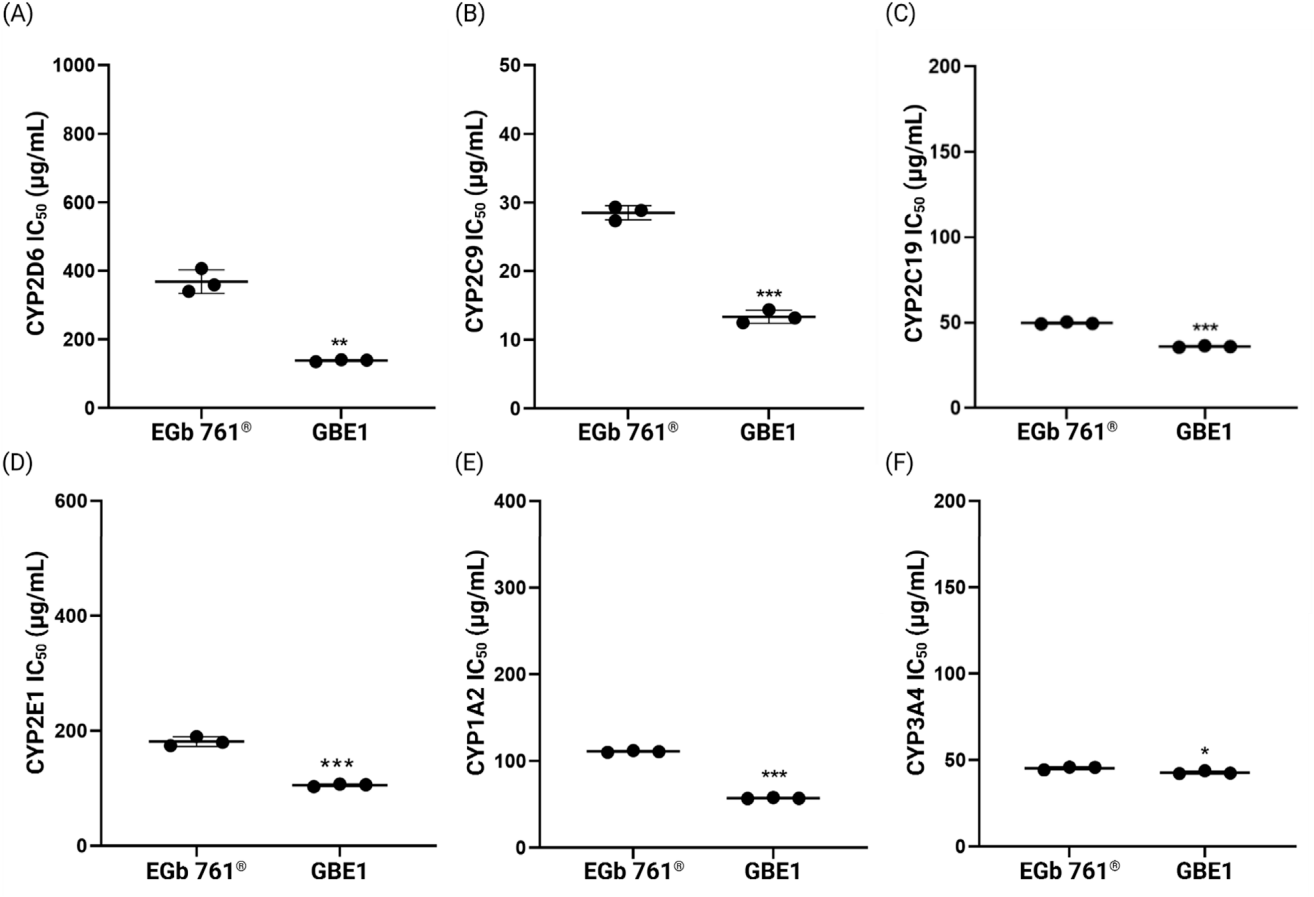
IC_50_ values for the inhibitory effects of raw materials EGb 761^®^ and GBE1 on the six representative CYP enzymes, **(A)** CYP2D6, **(B)** CYP2C9, **(C)** CYP2C19, **(D)** CYP2E1, **(E)** CYP1A2, and **(F)** CYP3A4 (*p < 0.05, **p < 0.01, ***p < 0.001 compared to EGb 761^®^, *t*-test).

Figure 7 illustrates the IC_50_ values of the CYP inhibitory activity measured for raw materials EGb 761^®^ and GBE1. Across all CYP enzymes, EGb 761^®^ consistently exhibited higher IC_50_ values compared to raw material B, indicating lower CYP inhibitory activity. The most significant difference was observed in the inhibitory activity against CYP2D6, where the average IC_50_ values were 366.6 ± 34.42 μg/mL for EGb 761^®^ and 138.4 ± 2.91 μg/mL for GBE1, representing a difference of approximately 62%.

**FIGURE 8:**
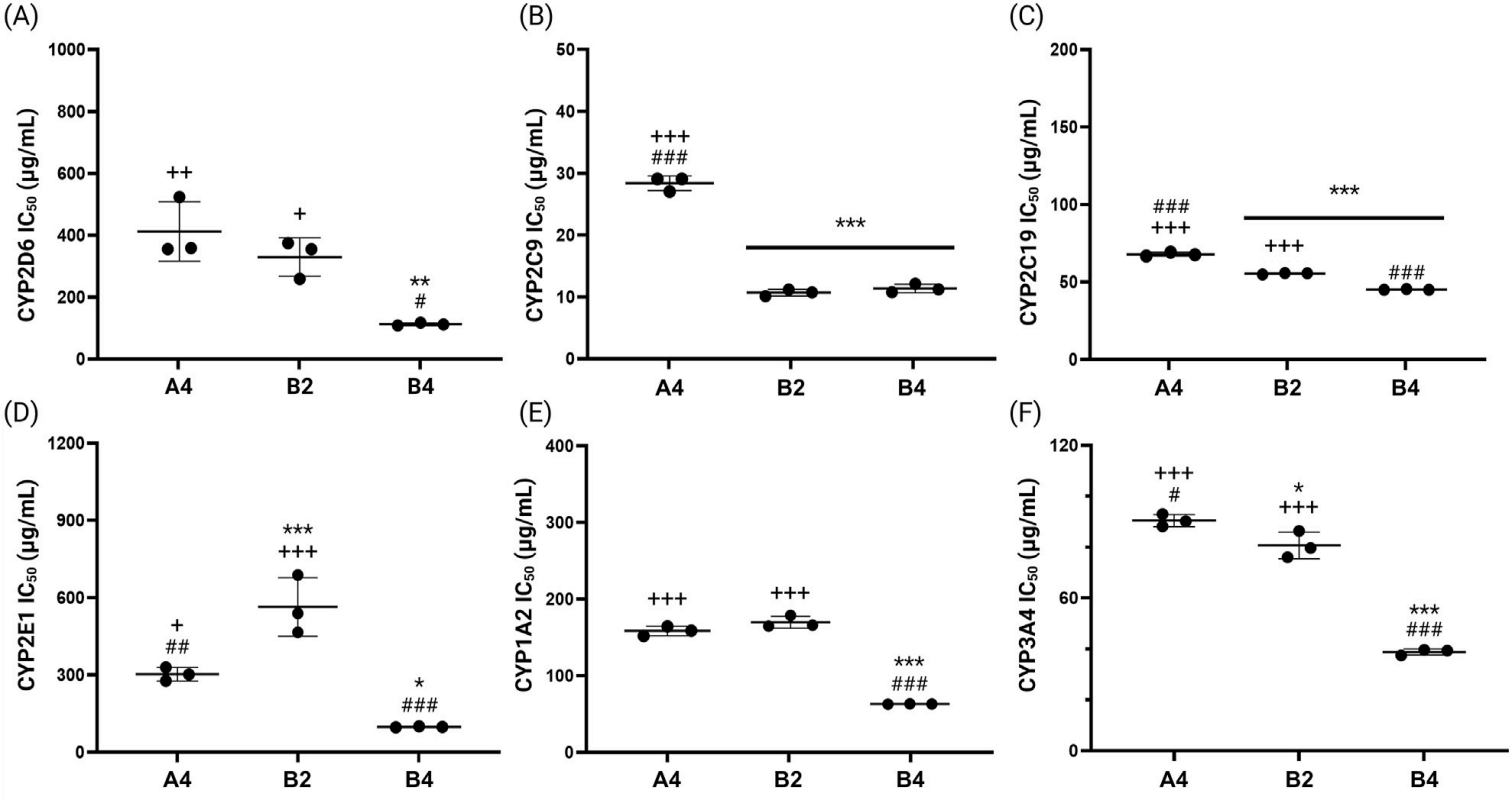
IC_50_ values for the inhibitory effects of final drug products (A4, B2 and B4), on the six representative CYP enzymes, **(A)** CYP2D6, **(B)** CYP2C9, **(C)** CYP2C19, **(D)** CYP2E1, **(E)** CYP1A2, and **(F)** CYP3A4 (*p < 0.05, **p < 0.01, ***p < 0.001 compared to A4; ^#^ *p* < 0.05, ^##^ *p* < 0.01, ^###^ *p* < 0.001 compared to B2; ^+^ *p* < 0.05, ^++^ *p* < 0.01, ^+++^ *p* < 0.001 compared to B4).

The IC_50_ values of the CYP inhibitory activity for the final drug products were also measured (Figure 8). Drug A4, manufactured from EGb 761^®^, showed the highest IC_50_ values for most CYP enzymes, demonstrating the lowest inhibitory activity against the tested CYP enzymes. Exceptionally, highest IC_50_ values were observed for B2 in case of CYP2E1, and drugs A4 and B2 both displayed comparatively high IC_50_ values (158.2 ± 5.91 μg/mL and 163.7 ± 7.86 μg/mL for A4 and B2, respectively) for CYP1A2. Drug B4, produced from GBE1, generally exhibited the lowest IC_50_ values, demonstrating the highest inhibitory activity against the tested CYP enzymes. These findings collectively indicate that EGb 761^®^ and drug A4 exhibit low inhibitory activity against key CYP enzymes, suggesting they are less likely to interfere with drug metabolism when used alongside other medications. Consequently, EGb 761^®^ and medications produced from this raw material are considered more appropriate for co-administration.

## Conclusion

In this study, we investigated the chemical composition differences of *G. biloba* leaf extracts, including EGb 761^®^, and the final drug products derived from these raw materials using HPLC and principal component analyses. The results revealed that EGb 761^®^ and its associated drug products (A1–A4) possess unique compositional characteristics that distinguish them from other raw materials and final products, while also maintaining consistent quality. Among the unique components, PCA was identified as a primary factor. The PCA content per *G. biloba* leaf extract was quantified across raw materials and drug products, showing higher levels in EGb 761^®^ and A1–A4, in agreement with prior findings. Molecular docking studies were conducted to evaluate the interactions between PCA and key proteins associated with cognitive function. The results demonstrated that PCA exhibited generally high binding affinities and stable binding energies with these proteins, which have previously been implicated in cognitive enhancement. Notably, PCA showed strong affinities toward M_1_ receptor, AChE, and GSK-3. These findings provide supportive evidence for the cognitive-enhancing effects of PCA reported in earlier studies and offer valuable insight for guiding future investigations into its underlying mechanisms of activity. Additionally, to assess the safety of co-administration, CYP inhibitory activity was evaluated on samples with high PCA content (EGb 761^®^, A4), low PCA content (GBE1, B4), and high PCA content but originating from a different raw material, GBE3 (B2). EGb 761^®^ exhibited significantly lower CYP inhibitory activity compared to GBE1, and A4 generally exhibited a tendency toward lower CYP inhibitory activity compared to B2 and B4. These findings suggest that EGb 761^®^ and its drug products may be more suitable for co-administration with a broader range of drugs compared to products derived from other raw materials. While a direct causal relationship between reduced CYP inhibitory activity and PCA could not be definitively confirmed–due to the presence of numerous other compounds in EGb 761^®^ and other raw materials or final drug products–further research is needed to clarify this relationship, as the chemical composition of *G. biloba* extracts is well-documented. This research underscores the importance of raw material selection in the development of superior cognitive-enhancing drugs and provides a foundation for expanding the recognized active components of *G. biloba* leaf extracts beyond flavonoid glycosides and terpenoid lactones to include organic acids such as PCA.

## Data Availability

Information for existing publicly accessible datasets is contained within the article.
