# Corrigendum: Optimized *Ginkgo biloba* extract EGb 761^®^: boosted therapeutic benefits with minimized CYP enzyme interference

**DOI:** 10.3389/jpps.2026.15563

**Published:** 2026-02-17

**Authors:** Sunbeom Kwon, Suji Jeong, Seulah Lee

**Affiliations:** 1 Department of Convergent Biotechnology and Advanced Materials Science, College of Life Sciences, Kyung Hee University, Yongin, Republic of Korea; 2 BK21 Interdisciplinary Program in IT-Bio Convergence System, Kyung Hee University, Yongin, Republic of Korea

**Keywords:** EGb 761^®^, protocatechuic acid, cognitive enhancement, molecular docking, CYP inhibition

In the published article, references 27 and 28 were cited in the **Conclusion** section. Due to the text correction listed below, these citations have been removed.

In the published article, there is an erroneous statement in the **Conclusion** section: “The potential inhibitory effects of PCA on CYP1A2, CYP2E1, CYP2D6, and CYP3A4 have been reported based on molecular docking simulations [27]. In particular, *in vivo* animal studies have demonstrated that PCA inhibits the enzymatic activity of CYP1A2 and CYP2E1 [28]. On the basis of prior research, it is reasonable to hypothesize that the enhanced CYP inhibition observed in EGb 761®, A4, and B2 is associated with their high concentrations of PCA.”

The correct statement is “While a direct causal relationship between reduced CYP inhibitory activity and PCA could not be definitively confirmed–due to the presence of numerous other compounds in EGb 761® and other raw materials or final drug products–further research is needed to clarify this relationship, as the chemical composition of *G. biloba* extracts is well-documented.”

The authors apologize for this error and state that this does not change the scientific conclusions of the article in any way. The original article has been updated.

