# Supplementary material for: Optimized Ginkgo biloba extract EGb 761®: boosted therapeutic benefits with minimized CYP enzyme interference
Source: J Pharm Pharm Sci. 2025 Aug 26;28:14614. doi: 10.3389/jpps.2025.14614 (PMC12417213; doi:10.3389/jpps.2025.14614)
Supplement: Supplementary file 1 [file Supplementaryfile1.docx]

Supplementary Material

**Table of Contents**

**Figure S1.** HPLC chromatograms of (A) PCA standard, (B) EGb 761^®^ and (C) A4…………3

**Figure S2.** EIC scan spectra (negative mode) of (A) PCA standard and (B) A4……..…………4

**Figure S3.** UV chromatograms of (A) PCA standard, (B) EGb 761^®^ and (C) A4...……………4

**Figure S4.** MS spectrum (negative mode) and calculated elemental composition of (A) PCA standard and (B) EGb 761^®^……………………………………………………………………5

**Figure S5.** Isotropic distribution pattern of (A) PCA standard and (B) EGb 761^®^…………5

**Figure S6.** MS/MS fragmentation profile of (A) PCA standard and (B) EGb 761^®^…………6

**Figure S7.** Optimized chromatograms for separation of PCA peak (A) PCA standard, (B) EGb 761^®^ and (C) A4………………………………………………………………………………7


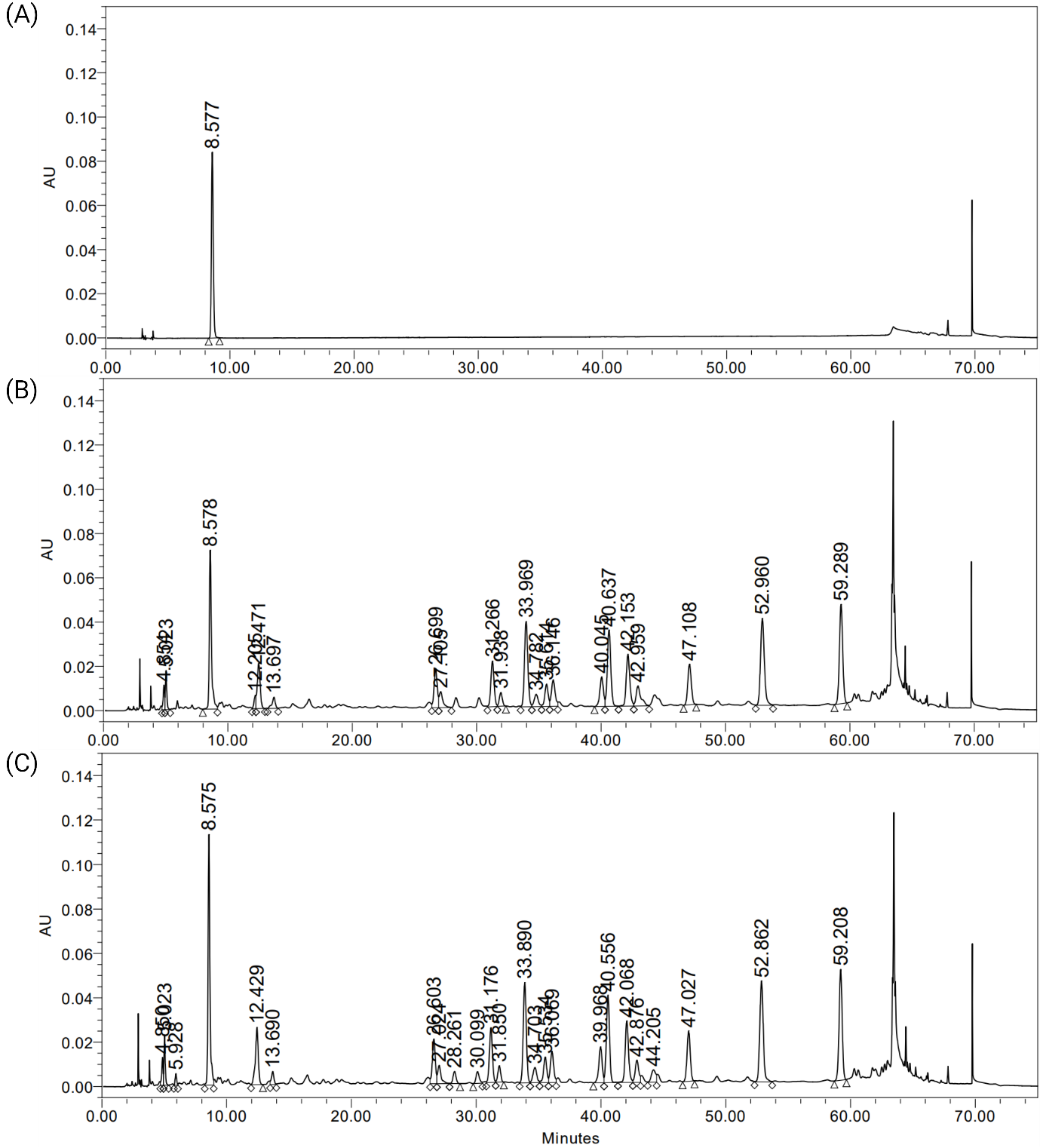
**Figure S1.** HPLC chromatograms of (A) PCA standard, (B) EGb 761^®^ and (C) A4.


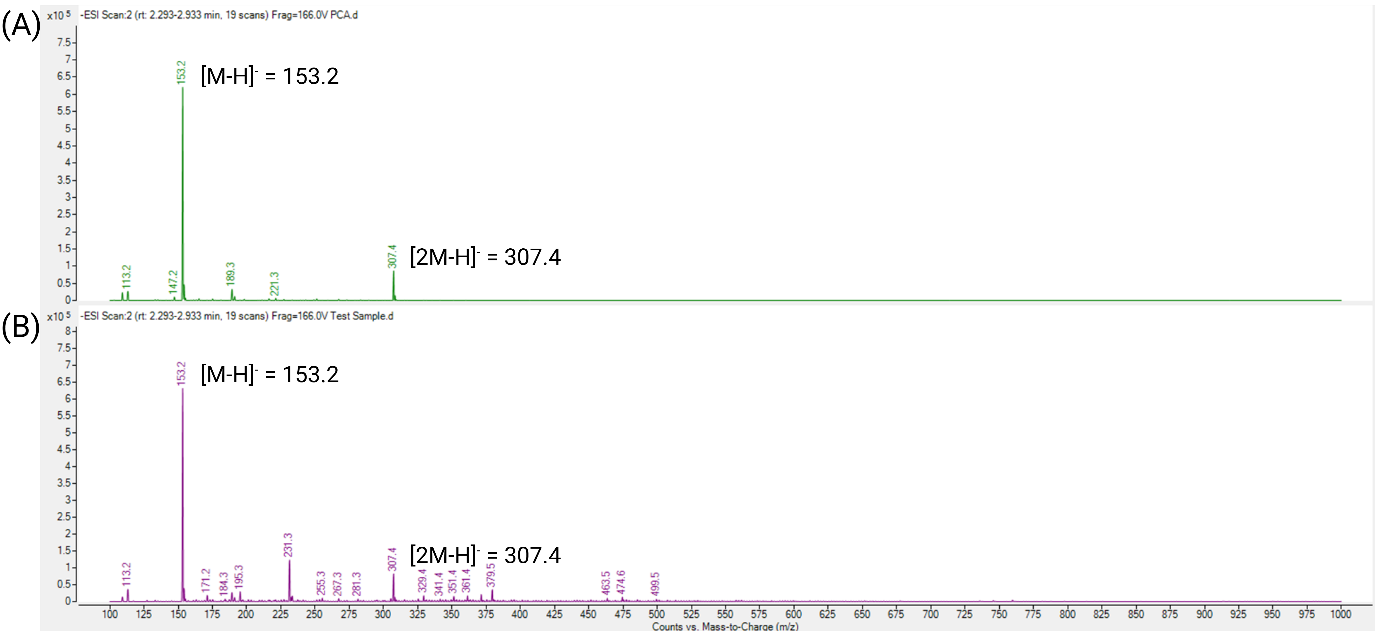


**Figure S2.** EIC scan (negative mode) of (A) PCA standard and (B) A4.


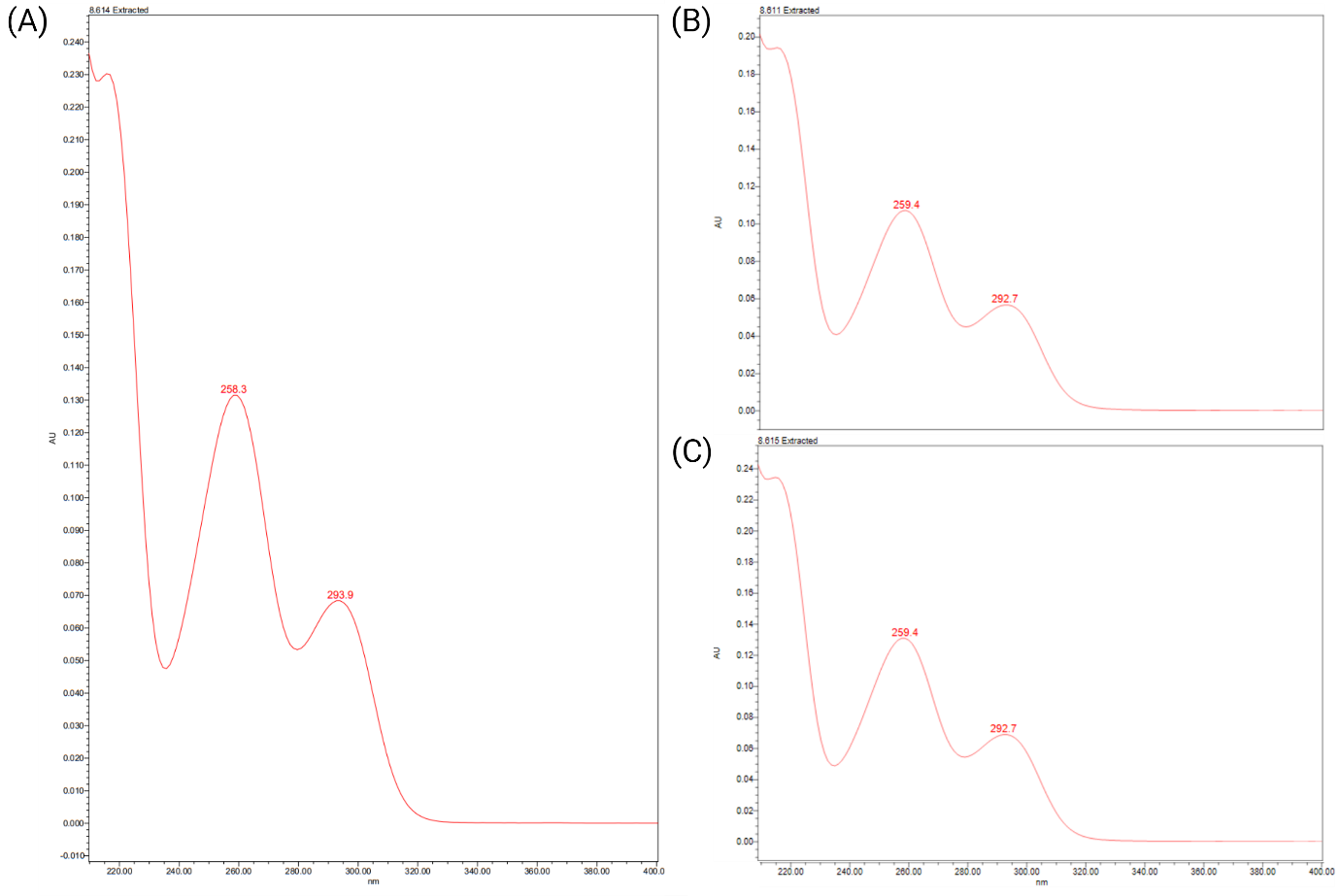


**Figure S3.** UV chromatograms of (A) PCA standard, (B) EGb 761^®^ and (C) A4.


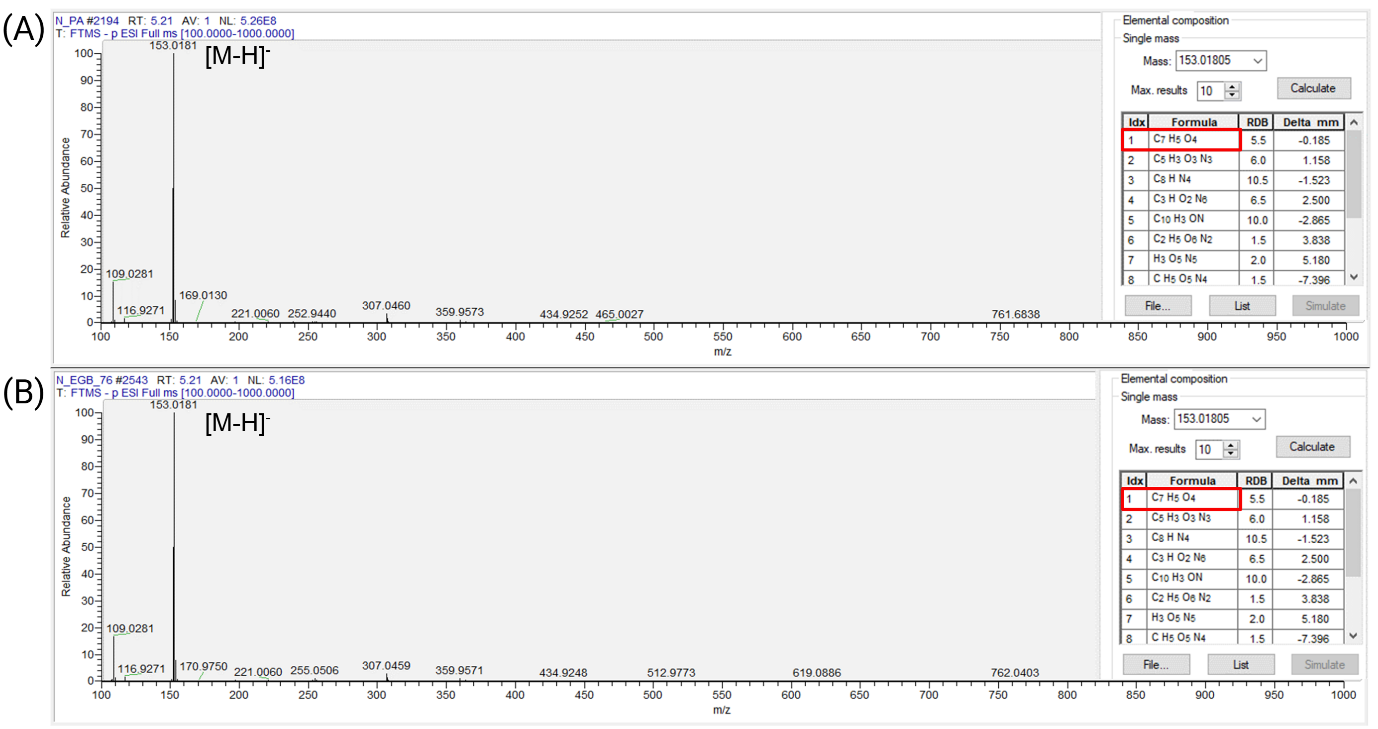


**Figure S4.** MS spectrum (negative mode) and calculated elemental composition of (A) PCA standard and (B) EGb 761^®^


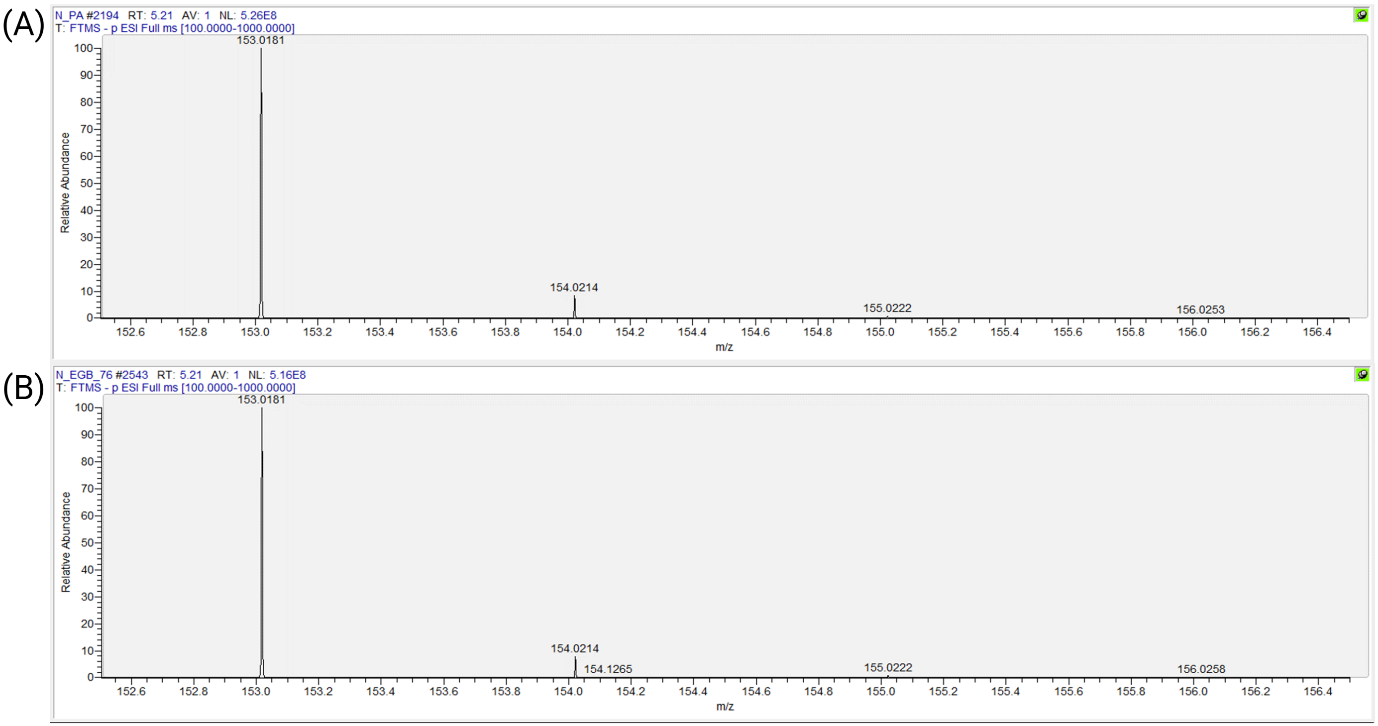


**Figure S5.** Isotropic distribution pattern of (A) PCA standard and (B) EGb 761^®^


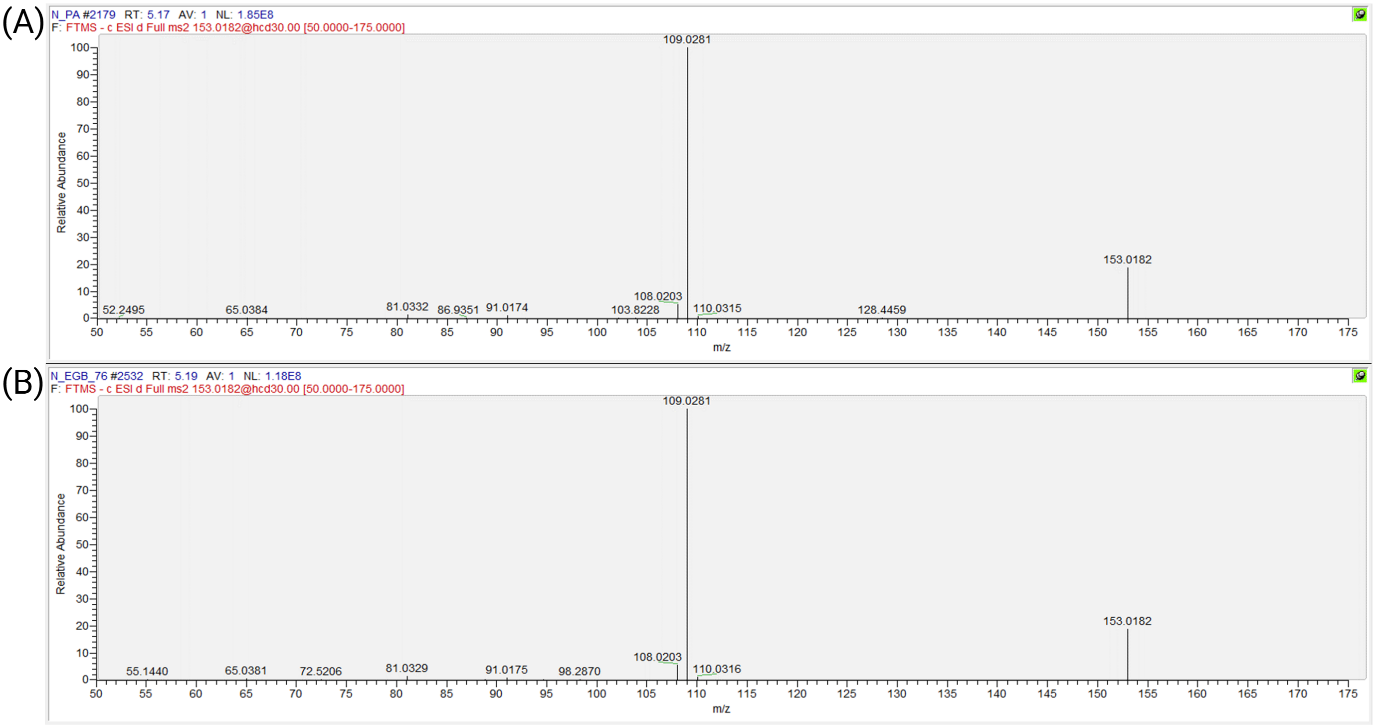


**Figure S6.** MS/MS fragmentation profile of (A) PCA standard and (B) EGb 761^®^


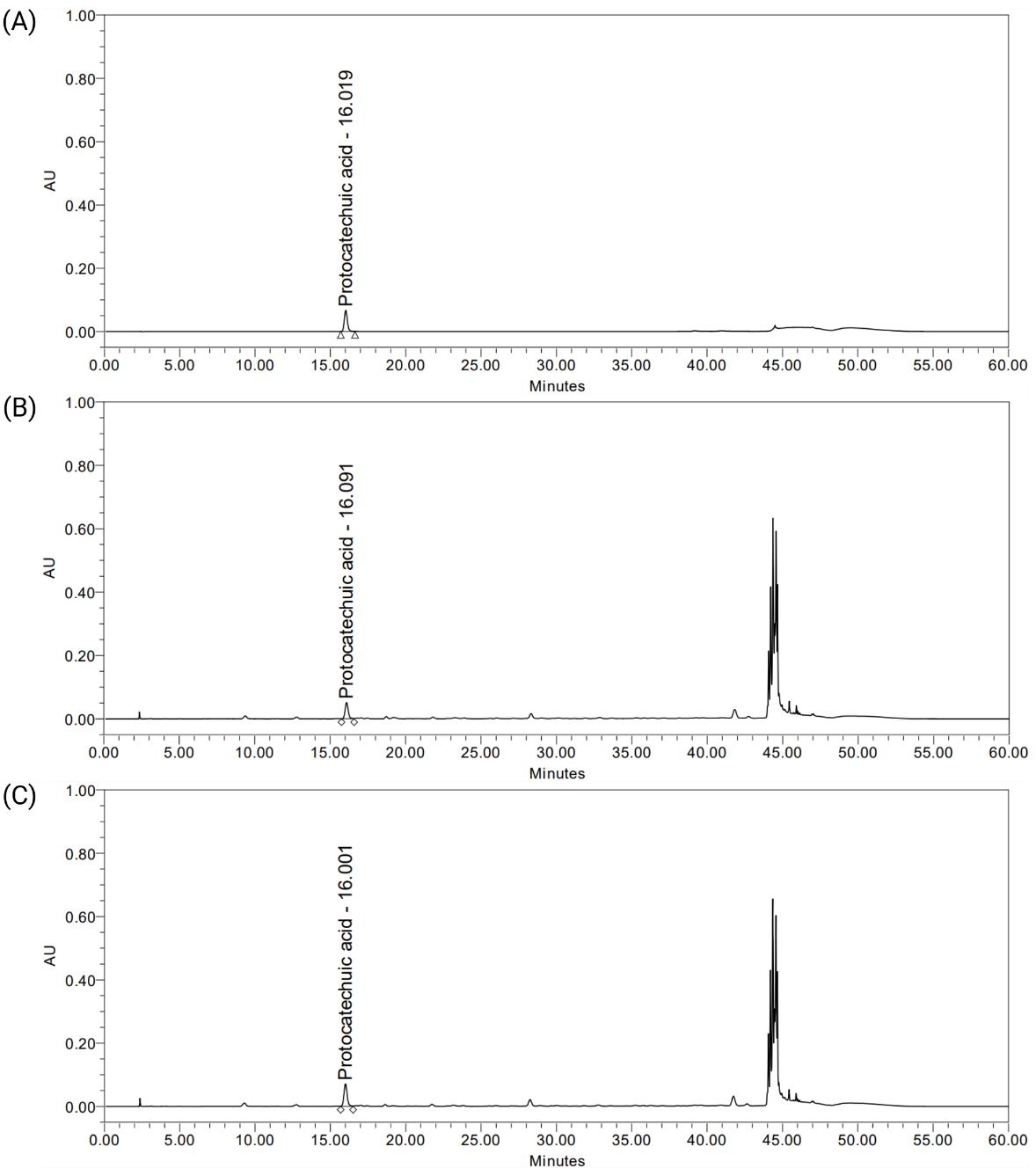


**Figure S7.** Optimized chromatograms for separation of PCA peak (A) PCA standard, (B) EGb 761^®^ and (C) A4
